# The role of lipid peroxidation products and antioxidant enzymes in the pathogenesis of aseptic and purulent inflammation in cats

**DOI:** 10.5455/javar.2021.h504

**Published:** 2021-06-17

**Authors:** Pavel Rudenko, Yuriy Vatnikov, Sergey Engashev, Andrey Kvochko, Elena Notina, Irina Bykova, Evgeny Kulikov, Andrey Rudenko, Olesia Petrukhina, Viktoriya Rudenko

**Affiliations:** 1 Biological Testing Laboratory, Branch of Shemyakin-Ovchinnikov Institute of Bioorganic Chemistry of the Russian Academy of Sciences, Moscow, Russia; 2 Department of Veterinary Medicine, Peoples’ Friendship University of Russia, Moscow, Russia; 3 Department of Parasitology and Veterinary and Sanitary Expertise, Moscow State Academy of Veterinary Medicine and Biotechnology, Moscow, Russia; 4 Department of Physiology, Surgery and Obstetrics, Stavropol State Agrarian University, Stavropol, Russia; 5 Deaprtment of Foreign Languages, Peoples’ Friendship University of Russia, Moscow, Russia

**Keywords:** Antioxidants, cats, inflammation, free radicals, ovariohysterectomy, respiratory burst, surgical infection

## Abstract

**Objective:**

The work aimed to determine the state of lipid peroxidation products and the activity of the antioxidant system in cats with aseptic inflammation and purulent-inflammatory processes of varying severity.

**Materials and Methods:**

The intensity of the process of lipid peroxidation- antioxidant system processes in blood plasma was evaluated using commercial kits. The level of diene conjugates (DC), the content of malondialdehyde (MDA), the level of medium-weight molecules were determined from lipid peroxidation indices. The state of antioxidant protection was assessed by indicators of superoxide dismutase (SOD) activity, catalase (CT), ceruloplasmin (CP) concentration, glutathione peroxidase activity, glutathione reductase (GLR), and total antioxidant activity (AOA).

**Results:**

With aseptic inflammation in the blood of cats, a slow activation of peroxidation reactions occurred. The DC indicator increased by 1.4 times, the SOD level rose by 1.38 times, the amount of GLR by 1.04 times, and the activation of total AOA by 9.06. In sepsis, the values of DC, MDA, and medium-weight molecules increased by 4.4, 8.4, and 8.8 times, respectively. With abscesses in the blood of cats, an increase in CT, CP, and GLR is observed against a background of a decrease in glutathione peroxidase. With purulent wounds, the total AOA of plasma increases by 2.5 times; with abscesses, the total AOA increases by 1.9 times, and with sepsis, it decreases by 1.5 times.

**Conclusion:**

With surgical infections in cats, deep biochemical changes or irreversible biochemical changes (in sepsis) occur that indicate a significantly enhanced catabolic orientation of metabolic processes and the accumulation of toxic metabolites, which leads to damage and a decrease in tissue regenerative capacity.

## Introduction

Ensuring reliable food security of the country in global deurbanization and deglobalization is currently one of the most important and prioritized national tasks. Therefore, the tasks set by veterinary medicine—namely, optimizing veterinary services and reducing morbidity and mortality of animals play a decisive role both in improving the quality of life of animals and in preserving the health of the entire human population as a whole [1-7]. The population of small domestic animals, including cats, is growing every year; the number of shelters and nurseries is increasing; the number of veterinary clinics is growing, new breeds of cats are being selected and imported from abroad. In the practice of treating members of the feline family, including cats, purulent-inflammatory processes of soft tissues hold one of the top places. The issues of formation, progression, and prediction of the course of purulent-inflammatory processes in members of the feline family, as well as their adequate treatment and prevention, remain under-researched [8–10].

Treatment of purulent-inflammatory processes to this day remains one of the most challenging and relevant problems in surgery. Over the past decade, increased purulent-inflammatory diseases and postoperative infectious complications in surgical patients have been recorded [11-16]. Despite the introduction of modern methods of treatment, the discovery of new generations of antibacterial agents, the use in pharmacological regimens of a significant arsenal of pharmacological agents with antimicrobial, necrolytic, analgesic, stimulating, and sorption effects, the continuous improvement of aseptic and antiseptic methods; despite all of this, the number of cases of complications of surgical infection has not decreased but rather increased. This leads to search the newer, more effective methods of combating purulent-inflammatory processes of soft tissues [8,10,17].

All biochemical, immunological, endocrine, and other processes in the body are directly or indirectly associated with the structure and function of biological membranes. Damage to cell membranes is one of the triggers for the development of many pathological processes. The basis of damage to cell membranes is the peroxidation process or lipid peroxidation (POL), free radical oxidation. POL, together with other toxic metabolites and inflammatory mediators, has a damaging effect on cells of organs and tissues. The antioxidant system (AOS) is one of the indicators of changes in the body. During the accumulation of reactive oxygen species (ROS) or a malfunction in AOS, the processes of lipid peroxidation and oxidative modification of proteins and nucleic acids are enhanced, which critically affects both the state of the cell itself and the state of the whole organism [11,18-25].

The wounding process is understood as a complex set of biological reactions that occur in the body, which develop in response to damage to the soft tissues and heal them. The nature of local reactions in a purulent wound is determined by the interaction of two damaging factors—the association of pathogens of surgical infection and the focus of tissue destruction. Local reactions in a purulent wound process are accompanied by the release of inflammatory mediators, various pathogenicity factors of microorganisms (the type of pathogens, their degree of sensitivity to antibiotics, the level of microbial contamination per 1 gm of tissue, etc.), resulting in damage to cells and tissues in the pathological focus. All these lead to violations of local microcirculation, metabolic processes in the wound tissues, and changes in its cellular composition [10,26-28].

As a result of disturbances in microcirculation in near-wound tissues, the oxygen content decreases. Hypoxia leads to inhibition of energy metabolism due to the break of the Krebs cycle. As a result, glycogen content in the cells (including neutrophilic granulocytes and lymphocytes) decreases. The energy deficit at the cellular level contributes to the development of purulent wound complications [8,9,29-33].

A significant role in the pathogenesis of purulent wounds must be assigned to the free radical mechanisms of tissue damage. Free radicals (oxidants) are molecular particles with an unpaired electron on the outer orbital and are one of the universal and critical mechanisms of cell damage and death. Free radicals and other toxic metabolites, together with inflammatory mediators, have an irreversible damaging effect on cells of organs and tissues by peroxidation of the lipid membrane of the cell membrane and organelles and by denaturation of enzymes, structural proteins, and denaturalization of the nucleus and polysaccharide complex of the interstitium of the basement membrane [27,30,34]. In addition to the usual molecular oxygen, which is in the triplet state of O2(3Σg—), there are six of its active derivatives. They include atomic oxygen—O, ozone—O3, singlet oxygen—O2(1Δg), superoxide radical—OO-, hydroxyl radical—OH, and hydroperoxide radical—OOH. Their high chemical activity characterizes free radicals, reaction with proteins, nucleic acids, lipids, destruction of cellular structures, and contribution to the formation of highly toxic products of free radical oxidation—peroxides, aldehydes, ketones [19,21,23,35].

The POL reactions is free radical and constantly occurs in the body. Free radical oxidation disrupts the structure of many molecules in the body, especially during the development of severe pathological conditions. The most significant products of POL processes are the level of diene conjugates (DC), the content of malondialdehyde (MDA), as well as the level of medium-weight molecules (MSM) [22,26].

Damage to membrane structures is prevented due to the presence in the cells of antioxidant defense (AOD), which consists of two subsystems—enzymatic and non-enzymatic [25]. The function of antioxidant enzymes in the body is to maintain a constant concentration of oxygen-containing radicals. Antioxidant enzymes include superoxide dismutase (SOD), catalase (CT), glutathione peroxidase (GLP), glutathione transferase, and ceruloplasmin (CP). All of them catalyze chemical reactions, resulting from which toxic free radicals and peroxides turn into compounds not harmful to the body. A critical point in the efficiency of the AOS enzymatic link is the balance of SOD, CT, and peroxidase. Inhibition of the activity of one of the enzymes can lead to excessive accumulation of ROS and destruction of cells. Under various pathological conditions, the concentration and activity of AOS enzymes can vary in different directions. The non-enzymatic component of AOS includes natural antioxidants: fat-soluble—vitamin E, β-Carotene, ubiquinones, and water-soluble—ascorbate, rutin, glutathione, which significantly inhibit substrate oxidation. The primary substrate in red blood cells is unsaturated fatty acids of cell membranes [18,20,36]. A progressive increase in tissue concentration of substances with pronounced antioxidant properties in specific conditions can contribute to the activation of adverse reactions by synthesizing toxic metabolites, prooxidants, and even ROS. We can assume that the combination of low molecular weight water-soluble compounds of this type regulates the intensity of free radical processes in tissues and promotes their participation in other metabolic pathways [11,23,37-40].

Thus, the study of indicators of POL-AOS processes, the dynamics of their changes during any inflammatory process, and targeted correction of the revealed violations can have diagnostic and prognostic value, serve as a pathophysiological justification for complex therapy, and will improve the final results of treatment. In this work, an attempt—summarizing our studies and the information available in the literature—is made to reflect the current state of the role in the pathogenesis of various forms of surgical infection in cats of lipid peroxidation and AOD of the body. Based on the preceding, the goal of our work was to determine the state of lipid peroxidation products and the activity of the antioxidant system in cats with aseptic inflammation, as well as with purulent-inflammatory processes of varying severity.

## Materials and Methods

Animal handling was led in accordance with legislation and international bioethical norms, the provisions of IV of the European Convention for the Protection of Vertebrate Animals used for Experimental and Other Scientific Purposes European treaty series 123 (1986), and after a favorable decision on bioethical Commission of the Lugansk National Agrarian University.

The material for the study was healthy cats, which were kept in a shelter for homeless animals of Yasinovatsky Machine-Building Plant closed joint-stock company in the city of Yasinovataya, Donetsk Region, and was determined in 21 healthy cats (10 males and 11 females). The age of the animals ranged from 2 to 5 years. The maximum and minimum values of the series, the arithmetic mean (M ± m), and the confidence interval (CI) calculated for *p* < 0.001 using the formula M ± m for *n* = 21 CI were determined. The state of the antioxidant system and the level of lipid peroxidation were also studied in clinically healthy cats who underwent planned surgery—ovariohysterectomy (*n* = 7), as well as in animals with random purulent wounds (*n* = 7), abscesses (*n* = 7), and also surgical diseases, which were accompanied by the development of sepsis (*n* = 7).

Blood sampling was performed by venous catheterization using a Venflon catheter. A pink Venflon™ Pro catheter (size 20G, length 32 mm, outer diameter 1.1 mm) was used. Blood samples from healthy animals were taken in the morning from cats that underwent ovariohysterectomy, not only immediately before the operation but also on the 3rd and 10th days in the postoperative period. Samples were also taken from patients with random forms of surgical infection when their initial admission to veterinary clinics. After plasma samples were centrifuged, they were frozen at −80°C and stored until analysis time. Blood samples of the control groups were processed similarly using the same methods and procedures. All samples were labeled with cat registration numbers to identify and prevent confusion.

Samples were examined using a spectrophotometer (UN2CO-WFT2100, Shanghai, China). Measuring lipid peroxidation products (the POL-AOS processes) in blood plasma was evaluated using commercial kits (RANDOX Laboratories Ltd., London, UK), according to the manufacturer’s instructions. Moreover, the level of diene conjugates (DC), the content of malondialdehyde (MDA), as well as the level of MSM were determined from lipid peroxidation indices. Measuring activity of the antioxidant system was assessed by indicators of SOD activity, CT, CP concentration, GLP activity, glutathione reductase (GLR), as well as total antioxidant activity (AOA).

Statistical analysis was processed on a personal computer using the statistical program STATISTICA 7.0 (StatSoft, Tulsa, OK) and presented in tables and figures. When conducting statistical calculations, the normality of the distribution was preliminarily evaluated using Shapiro–Wilk tests. In the case of a normal distribution of quantitative variables, Student’s *t*-test for independent samples was used to compare the two groups. The differences were considered significant at *p* < 0.05.

## Results and Discussion

The problem of indicators of the norm of the morpho-biochemical composition of the blood of domestic cats is more complex than it seems at first glance. If a significant number of publications are devoted to this problem when it comes to dogs, then about cats, one might say that the available data is quite limited. In addition, different authors cite ambiguous digital data, and this is understandable since studies are carried out in other conditions of keeping animals, and indicators depend on factors such as age, gender, diet composition, breed, etc. [1,5,8,10,11]. Therefore, when performing this work, we decided to examine clinically healthy cats kept in the conditions of the Lugansk and Donetsk regions, without clinical signs of any pathology, to create our control groups. Indicators of prooxidant-antioxidant homeostasis in clinically healthy cats are shown below in Table 1.

It was established that the confidence ranges for the DC parameter were 0.018–0.032 μmol; for MDA—0.86–1.38 μmol/l; for MSM—0.0042–0.0086 Conventional unit (C.U.); for CP—47.76–57.38 mg/l; SOD—0.29–0.43 C.U.; for CT—39.6–50.6 mkat/l; for GLP—18.54–23.34/min; for GLR—162.95–175.69 μmol/min; for AOA—24.45–36.05% (CI for *p* < 0.001).

Damage to the cell membrane, namely the phospholipid complex, is one of the triggers for developing many pathological processes. The primary role in the damage to these structures is played by POL processes, which, along with other toxic metabolites and inflammatory mediators, cause destruction of cell membranes, which leads to severe disorganization of the functions of organs and tissues of the body. This, in turn, is accompanied by suppression of protein synthesis and immune status [18,22,35,38,40].

**Table 1.** Indicators of prooxidant-antioxidant homeostasis in the blood of cats (*n* = 21).

Indicators	Lim	CI (*p* < 0,001)	M ± m
DC, μmol	0.012–0.048	0.018–0.032	0.025 ± 0.002
MDA, μmol/l	0.46–1.69	0.86–1.38	1.12 ± 0.07
MSM, C.U.	0.002–0.012	0.0042–0.0086	0.0064 ± 0.0006
CP, mg/l	43.50–64.60	47.76–57.38	52.57 ± 1.26
SOD, C.U.	0.24–0.59	0.29–0.43	0.36 ± 0.02
CT, mkat/l	36.28–58.65	39.6–50.6	45.10 ± 1.45
GLP, μmol/min	16.70–26.40	18.54–23.34	20.94 ± 0.63
GLR, μmol/min	153.40–186.50	162.95–175.69	169.32 ± 1.67
AOA, %	19.05–46.24	24.45–36.05	30.25 ± 1.52

Therefore, we further studied the level of POL products and the activity of antioxidant enzymes in the blood of cats with an aseptic inflammatory process—a planned surgical intervention. The results are shown in Table 2.

The studies presented in Table 2 showed that with aseptic inflammation in the blood of cats on the 3rd day of the postoperative period, a slow activation of peroxidation reactions occurred. Thus, in animals, the DC indicator increased significantly (*p* < 0.05) by 1.4 times from 0.023 ± 0.003 to 0.035 ± 0.002 μmol, compared with the initial data. On the 10th day of the postoperative period, the lipid peroxidation index decreased and approached the values of clinically healthy animals.

The low level of free radicals in cells is controlled by the presence in the body of an AOS, the inhibitors of which can directly react with free radicals. Under physiological conditions, AOS protects cellular lipids from excessive peroxidation; therefore, it is considered one of the most significant indicators of homeostasis. Even short-term failure of AOS causes significant disturbances in homeostatic processes, and a longer existence of free radicals can lead to irreversible damage to organoids of cells and tissues [14,18-20,25]. It was found that, on the 3rd day after the application of an operating injury in the blood of animals, a significant increase in the SOD level occurred by 1.38 times from 0.34 ± 0.03 to 0.47 ± 0.03 C.U. (*p* < 0.01) and the amount of GLR 1.04 times from 170.70 ± 2.59 to 177.87 ± 1.71 μmol/min (*p* < 0.05) compared with clinically healthy cats. However, on day 10, with a decrease in the activity of aseptic inflammation, the level of antioxidant enzymes approached the initial values.

**Figure 1.**
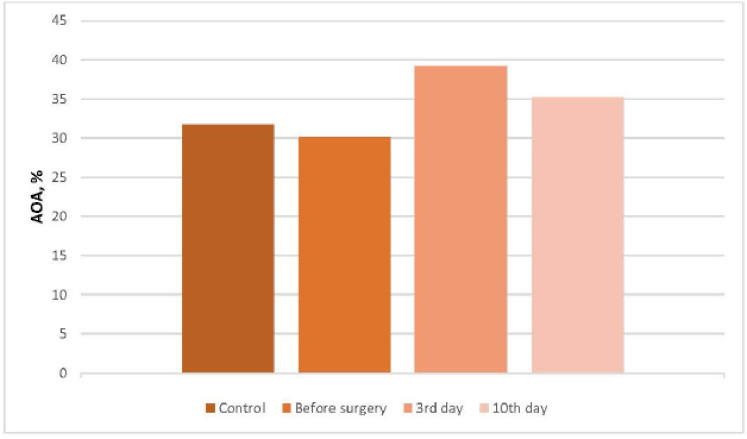
Total AOA of plasma in cats with an operational wound.

AOA of blood plasma is an integral indicator, which indicates the level of total protection of the body from toxic products of POL [3,5,21]. Total AOA of plasma in cats with an operational wound is shown in Figure 1.

**Table 2.** The level of lipid peroxidation products and the activity of antioxidant enzymes in the blood of cats with an operational wound.

Indicators	Control (*n* = 7)	Before surgery (*n* = 7)	Postoperative period (*n* = 7)	
			3^d^ day	10th day
DC, μmol	0.023 ± 0.003	0.025 ± 0.002	0.035 ± 0.002*(↑34.3%)	0.029 ± 0.002
MDA, μmol/l	1.15 ± 0.11	1.10 ± 0.12	1.47 ± 0.11	1.34 ± 0.11
MSM, C.U.	0.006 ± 0.001	0.007 ± 0.001	0.008 ± 0.001	0.008 ± 0.001
CP, mg/l	51.16 ± 2.15	52.11 ± 1.51	53.74 ± 1.49	54.40 ± 1.33
SOD, C.U.	0.34 ± 0.03	0.35 ± 0.02	0.47 ± 0.03**(↑27.6%)	0.37 ± 0.02
CT, mkat/l	43.78 ± 2.31	44.30 ± 2.33	48.98 ± 2.62	46.02 ± 2.44
GLP, μmol/minute	20.18 ± 1.07	20.57 ± 0.88	21.86 ± 0.76	21.47 ± 0.74
GLR, μmol/minute	170.70 ± 2.59	166.67 ± 2.01	177.87 ± 1.71*(↑3.9%)	174.08 ± 1.68

* *p* < 0.05;

** *p* < 0.01 in comparison with the control group.

With ovariohysterectomy in the blood of cats, activation of total AOA occurred. So, this indicator on the 3rd day of the postoperative period increased by 9.06%, from 30.11 ± 3.04 to 39.17 ± 2.65, and on the 10th day—by 5.48%, from 30.11 ± 3.04 to 35.59 ± 2.94, when compared with the initial data.

Thus, the state of the body and the physiological course of vital processes depended on the balance and stability of POL-AOS. That means that the state of the body and vital processes depended on the state and reserves of the bio-antioxidative system, on the one hand, and depended on the activity of the damaging factor that activates POL’s processes on the other.

Necessary in the scientific concept of the pathogenesis of severe forms of wound infection and purulent-inflammatory conditions are the processes of lipid peroxidation and the state of AOS, the relationship of which significantly determines the direction of the disease. The imbalance between these systems determines the nature, intensity of development, and prognosis of a purulent-inflammatory process [7,9,11,17]. Also, significant changes were observed in the indicators of lipid peroxidation products in the blood of cats with various forms of surgical infection, which are shown in Table 3.

The studies presented in the table shown that in case of random purulent-inflammatory processes in the blood of cats, peroxidation reactions occurred, which, depending on the severity of the surgical infection, deepens and reaches its maximum in the group of animals with sepsis. So, in cats with sepsis, DC, MDA, and MSM significantly increased by 4.4, 8.4, and 8.8 times (*p* < 0.001), respectively, compared with clinically healthy animals.

**Figure 2.**
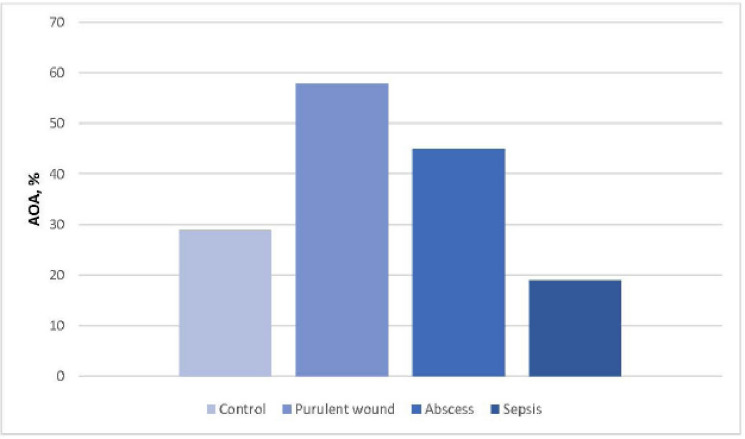
Total AOA of blood plasma in cats with various forms of surgical infection.

The results obtained indicated that the enzymatic link of AOD in cats with different forms of surgical infection was at a different level. So, with purulent wounds in the blood of animals, an increase in the level of all antioxidant enzymes occurred. With abscesses in the blood of cats, an increase in the indices of CT, CP, and GLR was observed against the background of a decrease in GLP from 22.07 ± 1.33 to 15.91 ± 0.95 μmol/min (*p* < 0.01). In our opinion, the data on the state of the AOD system in cats with sepsis are interesting. So, in animals, there was a highly reliable decrease in SOD, CT, GLP, and GLR (*p* < 0.001) at 2.37; 1.79; 2.47; 2.04 times, respectively, against the background of a significant (*p* < 0.001) increase in CP from 54.44 ± 2.85 to 104.80 ± 3.90 mg/l by 1.92 times when compared with the control group. This indicated a depletion of the AOD system. Total AOA of blood plasma of cats in case of random purulent-inflammatory processes is shown in Figure 2.

With various forms of surgical infection in cats, significant changes in the total AOA of plasma occurred. So, with purulent wounds, this indicator significantly increased by 2.5 times from 28.88 ± 2.77 to 57.81 ± 5.86 (*p* < 0.001), with abscesses, total AOA increased by 1.9 times, to 44.93 ± 3.30 (*p* < 0.01), and with sepsis, it decreased 1.5 times—to 18.86 ± 2.35 (*p* < 0.05) compared with clinically healthy animals.

Thus, during surgical infections in cats’ bodies, profoundly adverse and irreversible biochemical changes occurred that indicate a significantly enhanced catabolic orientation of metabolic processes and the accumulation of toxic metabolites, which lead to damage and a decrease in the regenerative capacity of tissues. Thus, the body of domestic cats exhibited significant resistance to the damaging effects of surgical infections with purulent wounds and abscesses, responding to microbial intervention with a typical reaction of acute phase inflammation. 

**Table 3.** The level of lipid peroxidation products and the activity of antioxidant enzymes in the blood of cats with surgical infection.

Indicators	Control(*n* = 7)	Random purulent-inflammatory processes		
		Purulent wounds (*n* = 7)	Abscesses (*n* = 7)	Sepsis (*n* = 7)
DC, μmol	0.027 ± 0.004	0.052 ± 0.003**(↑48.1%)	0.064 ± 0.003***(↑57.8%)	0.12 ± 0.02***(↑77.5%)
MDA, μmol/l	1.13 ± 0.16	2.87 ± 0.20***(↑60.6%)	4.55 ± 0.41***(↑75.2%)	9.50 ± 0.47***(↑88.1%)
MSM, C.U.	0.006 ± 0.001	0.015 ± 0.002**(↑60.0%)	0.024 ± 0.002***(↑75.0%)	0.053 ± 0.006***(↑88.7%)
CP, mg/l	54.44 ± 2.85	70.47 ± 4.01**(↑22.7%)	83.65 ± 3.16***(↑34.9%)	104.80 ± 3.90***(↑48.0%)
SOD, C.U.	0,38 ± 0.04	0.74 ± 0.04***(↑48.6%)	0.38 ± 0.03	0.16 ± 0.02***(↓57.9%)
CT, mkat/l	47.22 ± 3.06	53.29 ± 2.65	55.73 ± 2.87	26.27 ± 2.47***(↓44.4%)
GLP, μmol/minute	22.07 ± 1.33	24.87 ± 0.52	15.91 ± 0.95**(↓27.9%)	8.94 ± 0.81***(↓59.5%)
GLR, μmol/minute	170.61 ± 3.93	195.84 ± 4.91**(↑12.9%)	207.13 ± 7.25***(↑17.6%)	83.41 ± 3.57***(↓51.1%)

** *p* < 0.01;

*** *p* < 0.001 in comparison with the control group.

## Conclusion

Thus, the state of the body and the physiological course of life processes depend on the balance and stability of POL-AOS, that is, on the state and reserves of the bio-antioxidative system, on the one hand, and also on the activity of a damaging factor that activates the POL processes, on the other. The imbalance between these systems determines the nature, intensity of development, and prognosis of a purulent-inflammatory process. A detailed study of the indicators of POL-AOS processes in purulent-inflammatory pathologies in cats can have a diagnostic and prognostic value for the course of any inflammatory process, serve as a justification for the use of one or another protocol of complex therapy, which will undoubtedly improve the final results of treatment.

## List of Abbreviations

Antioxidant activity, AOA; Antioxidant defense, AOD; Arithmetic mean, M ± m; Catalase, CT; Ceruloplasmin, CP; Confidence interval, CI; Conventional unit, C.U.; Diene conjugates, DC; Glutathione peroxidase, GLP; Glutathione reductase, GLR; gram, gm; Malondialdehyde, MDA; Medium-weight molecules, MSM; milligrams per liter, mg/l; millimeter, mm; millikatal per liter, mkat/l; Process of lipid peroxidation, POL; Reactive oxygen species, ROS; Superoxide dismutase, SOD; Total antioxidant system, AOS; micromoles per minutes, μmol/min; micromole, μmol; micromoles per liter, μmol/l.
